# Low molecular weight serum cell-free DNA concentration is associated with clinicopathologic indices of poor prognosis in women with uterine cancer

**DOI:** 10.1186/s12967-020-02493-8

**Published:** 2020-08-27

**Authors:** Gregory M. Gressel, Elaine C. Maggi, Bryan E. Harmon, Kenny Q. Ye, D. Y. S. Kuo, Siobhan M. Dolan, Cristina Montagna

**Affiliations:** 1 Department of Obstetrics & Gynecology and Women’s Health, Albert Einstein College of Medicine, Bronx, NY USA; 2 Department of Genetics, Albert Einstein College of Medicine Price Center/Block Research Pavilion, Room 401, 1301 Morris Park Avenue, Bronx, NY 10461 USA; 3 Department of Pathology, Albert Einstein College of Medicine, Bronx, NY USA; 4 Department of Epidemiology & Population Health, Albert Einstein College of Medicine, Bronx, NY USA; 5 Department of Systems & Computational Biology, Albert Einstein College of Medicine, Bronx, NY USA

**Keywords:** Cell-free DNA, Endometrial cancer, Uterine cancer, Serum biomarkers, Serum DNA

## Abstract

**Background:**

Serum cell-free DNA (cfDNA) holds promise as a non-invasive cancer biomarker. The objective of this study was to evaluate the association of cfDNA concentration with clinicopathologic variables of poor prognosis and overall survival among women with uterine cancer compared to benign cancer-free controls.

**Methods:**

cfDNA was extracted from the serum of 91 women with multiple uterine cancer histologies and 22 post-menopausal controls without cancer. Low molecular weight (LMW) cfDNA was separated from contaminating genomic high molecular weight cfDNA using paramagnetic bead purification and its concentration was measured using fluorometric quantification. Clinicopathologic data was abstracted from the electronic medical record. The association between serum cfDNA concentration, clinicopathologic variables, and overall survival was assessed using linear regression modelling, Cox proportional hazards modelling, and the Kaplan–Meier method.

**Results:**

Median total serum cfDNA concentration for the cohort was 69.2 ng/mL (IQR 37.4, 132.3) and median LMW cfDNA concentration was 23.8 ng/mL (IQR 14.9, 44.4). There were no significant differences in total serum cfDNA concentration with any clinicopathologic variables. However, LMW cfDNA concentration was significantly higher in serum of women with cancer (25.8 ng/mL IQR 16.0, 49.6) compared to benign controls (15.5 ng/mL IQR 9.3, 25.8 ng/mL) (p < 0.01). It is also significantly higher among women with early stage cancer than benign controls (p < 0.01). There were also significant associations between LMW cfDNA concentration and stage of cancer (p = 0.01) and histology (p = 0.02). Patients with leiomyosarcoma and carcinosarcoma had higher cfDNA concentrations than those with endometrioid cancer. Over a median follow-up of 51.9 months, 75th percentile for overall survival for women with cancer was 24.0 months. Higher LMW cfDNA concentrations is associated with lower survival among women with cancer (p < 0.01).

**Conclusions:**

Serum LMW cfDNA concentration is associated with overall survival in women with uterine cancer, and it is higher among women with uterine cancer compared to those of controls.

## Background

Uterine cancer is the most common gynecologic malignancy with 61,880 new cases and 12,160 deaths anticipated in the United States in 2019 [1]. Early detection of uterine cancer is associated with improved survival relative to detection at later stages of disease, especially for high-risk, high-grade histologies such as uterine papillary serous carcinoma (UPSC), clear cell carcinoma (CCC), carcinosarcoma (CS) and leiomyosarcoma (LMS) which represent less than 10% of uterine cancers, but are responsible for as many as 20–39% of uterine cancer-related deaths [2, 3]. Currently there are no available screening tests or reliable biomarkers for early detection of uterine cancer. Diagnosis is traditionally made using an endometrial biopsy which is a painful and invasive procedure associated with potential bleeding, infection and uterine perforation. As such, endometrial biopsy is not ideal for serial sampling, assessing response to therapy, or monitoring for disease progression. Thus, there exists an unmet need for a non-invasive serum test for early uterine cancer diagnosis and longitudinal follow-up, especially to aid in the identification of high-risk uterine cancer, which behaves aggressively and has a propensity to metastasize.

Increasing attention is now paid to less invasive methods for disease surveillance such as analysis of cell free tumor DNA (cfDNA). While cfDNA can be isolated from the serum or plasma of any individual, patients with cancer have higher circulating concentrations, largely as a result of tissue necrosis and apoptosis, leading to release of tumor DNA into the blood stream [4]. Liquid biopsies are advantageous when compared with traditional biopsy as they are cost-effective, less invasive, easily obtainable in an outpatient setting, and can be collected longitudinally to monitor progression and response to therapy. Liquid biopsies have also been used in other disease sites for early cancer detection, even before the development of clinical symptoms [5].

cfDNA is composed of short DNA fragments (~ 160 base pairs) and its multiples, roughly corresponding to the length of a nucleosome [6]. The major challenge of cfDNA analysis is that the cfDNA derived from tumor comprises only a small fraction of the total pool of cfDNA in the bloodstream, with non-tumor cfDNA being far more abundant [7]. Our laboratory has previously described methodology for purifying high molecular weight contaminating genomic DNA from tumor-related low molecular weight (LMW) cfDNA [8]. The association of LMW cfDNA concentration with clinical outcomes remains unclear and has been under-reported in uterine cancer [9]. The objective of the current study is to characterize the association of circulating LMW cfDNA concentration with clinicopathologic variables that portend a poor prognosis in uterine cancer. Our a priori hypothesis is that LMW cfDNA concentration will be higher in women with more aggressive disease and those with poor overall survival.

## Methods

### Cell-free DNA isolation, purification and quantification

Aliquots of serum (1–3 mL) were prospectively collected from 91 women diagnosed with uterine cancer immediately prior to surgical staging and from 22 post-menopausal controls without cancer undergoing surgery for benign indications. Approximately 10 mL of blood were collected and placed in red topped tubes and allowed to sit upright at room temperature for 30 min before processing. Serum was separated from red cells by centrifugation at 2500 RPM at 4 ^°^C. These samples were aliquoted in 1–3 mL vials, biobanked and frozen at − 80 ℃ as part of an ongoing tissue collection protocol as part of the Albert Einstein Gynecologic Cancer Tissue Repository. All collection and experimental procedures were approved by the Internal Review Board of the Albert Einstein College of Medicine (IRB# 2009-265, IRB# 2017-8292). The histologic type of each primary tumor specimen was reviewed by and confirmed by an expert gynecologic pathologist.

cfDNA was isolated from serum samples using the Qiagen QIAamp Circulating Nucleic Acid kit (Cat no./ID 55114) and a vacuum manifold according to the manufacturer’s protocol. After eluting the samples in the provided elution buffer, the concentration of total cfDNA per mL of serum was calculated using fluometric quantification (Qubit). Low-molecular weight tumor-related DNA (LMW cfDNA) was purified from contaminating high molecular weight genomic DNA using Solid Phase Reversible Immobilisation beads (SPRI) from Beckman Coulter (Agencourt AMPure cat#A63880) using methods previously described by our laboratory [8]. This DNA purification step has been demonstrated to remove contaminating traces of high-molecular weight genomic DNA (~ 10,380 bp) from tumor-derived DNA (~ 150–200 bp).

Briefly, two consecutive AMPure bead steps were used in the size-selection protocol: The first step used a 0.5 × bead to sample volumetric ratio to remove the large genomic DNA fragments. The second step used a 1.6× bead to sample volumetric dilution ratio in order to bind the desired cfDNA fragments. Final LMW cfDNA samples were eluted in low TE buffer and quantified using the Qubit. All samples were bioanalyzed using a high sensitivity DNA chip (Agilent, Santa Clara, CA, United States) to assess the distribution of cfDNA size.

### Analysis of clinicopathologic variables

Clinical records for benign controls and women with uterine cancer were reviewed by a physician. Demographic information including age, race, ethnicity and body mass index (kg/m^2^) were collected. For subjects with cancer, clinicopathologic variables were abstracted from the medical record including cancer histologic subtype, cancer stage, tumor size, percent myometrial invasion, and presence of lymphovascular space invasion (LVSI) or lymph node involvement. These variables have previously been examined in multiple large uterine cancer studies and are classically associated with poor prognosis from disease [10–12].

Data analysis was performed using Stata version 14.2 (StataCorp. 2015. Stata Statistical Software: Release 14. College Station, TX: StataCorp LP) and R (R Core Team (2017). R: A language and environment for statistical computing. R Foundation for Statistical Computing, Vienna, Austria). Normality of continuous variables was visually assessed, and, if no substantial violations were noted, data were reported as means ± standard deviations. Otherwise, they were reported as medians with interquartile ranges. The association between each clinicopathologic variable and cfDNA concentration was assessed using univariate statistical tests including Kruskal-Wallis test, Wilcoxon rank-sum test and Pearson correlation. The comparison between cfDNA concentration in individual cancer histologies and benign controls were performed using linear regression modelling with log-transformed cfDNA concentration as the response and age as a covariate. p-values are computed based on the one-sided tests. Overall survival at the 75th percentile was calculated from time of diagnosis to death from cancer or censorship. Patients lost-to follow-up, who died from other causes, or who were alive at last contact were censored from analysis. Cox regression was used to evaluate the association between quartiles of LMW cfDNA concentration and survival, with age as a covariate. As there is no a priori consensus about what constitutes a “high” versus “low” cfDNA concentration, quartiles of LMW cfDNA concentration were used in the analysis. The Kaplan–Meier method was used to evaluate the differences in overall survival among quartiles of LMW cfDNA concentration.

## Results

Demographic information for patients included in the study are presented in Table 1. The cohort was predominantly post-menopausal (median age 62.2 ± 11.7) and included racially and ethnically diverse women (50% non-white race, 32% Hispanic ethnicity). A broad range of uterine histologies was represented including all grades of endometrioid adenocarcinoma (EAC, N = 47), uterine papillary serous carcinoma (UPSC, N = 15), carcinosarcoma (CS, N = 15), leiomyosarcoma (LMS, N = 8), and clear cell carcinoma (CCC, N = 6). These patients had predominantly International Federation of Gynecology and Obstetrics (FIGO) stage I disease (45%) but over half of the cohort had more advanced disease (stage II–18%, stage III–24%, stage IV–13%) at the time of diagnosis. Patients with more aggressive tumor subtypes such as UPSC, CS, LMS and CCC generally had larger tumors, deeper myometrial invasion, and more frequent LVSI or lymph node involvement than those with EAC.

**Table 1:** Clinical and pathologic data of patients included in the study stratified by uterine cancer histologic subtype

	Total (N = 113)	Benign^a^ (N = 22)	G1 EAC (N = 16)	G2 EAC (N = 15)	G3 EAC (N = 16)	UPSC (N = 15)	CS (N = 15)	LMS (N = 8)	CCC (N = 6)
Age	62.2 ± 11.7	55.6 ± 13.1	65.0 ± 11.6	62.4 ± 9.2	63.4 ± 10.2	71.5 ± 5.7	65.5 ± 6.4	50.6 ± 12.1	58.3 ± 14.6
Race									
White	57 (50)	13 (59)	11 (69)	8 (53)	9 (56)	4 (27)	6 (40)	3 (38)	3 (50)
Black	49 (43)	7 (32)	5 (31)	6 (40)	3 (19)	11 (73)	9 (60)	5 (62)	3 (50)
Asian	7 (6)	2 (9)	0 (0)	1 (7)	4 (25)	0 (0)	0 (0)	0 (0)	0 (0)
Hispanic	36 (32)	5 (23)	6 (38)	7 (47)	6 (38)	2 (13)	5 (33)	3 (38)	2 (33)
Body mass index (kg/m^2^)	31.1 (24.2, 37.0)	31.1 (23.2, 38.8)	32.3 (27.7, 43.5)	33.4 (28.0, 42.1)	27.5 (21.6, 37.2)	26.1 (21.8, 33.8)	30.7 (22.3, 37.1)	27.4 (25.3, 33.5)	34.7 (27.5, 39.1)
Stage^b^									
I	41 (45)	–	10 (63)	11 (73)	8 (50)	1 (7)	3 (20)	3 (38)	5 (83)
II	16 (18)	–	4 (25)	2 (13)	1 (6)	6 (40)	1 (7)	1 (13)	1 (17)
III	22 (24)	–	0 (0)	2 (13)	7 (44)	6 (40)	6 (40)	1 (13)	0 (0)
IV	12 (13)	–	2 (13)	0 (0)	0 (0)	2 (13)	5 (33)	3 (38)	0 (0)
Tumor size (cm)^b^	5.5 (3.5, 8.7)	–	3.9 (2.7, 5.2)	4.5 (3.5, 9.0)	5.3 (3.3, 8.0)	7.5 (5.2, 9.0)	8.5 (4.8, 10.0)	9.5 (5.8, 12.3)	4.5 (3.5, 6.0)
Percent myometrial invasion^b^	33.0 (12.5, 79.0)	–	28.5 (13.5, 51.5)	21.0 (10.0, 45.0)	57.5 (22.0, 88.8)	61.0 (13.0, 87.0)	18.0 (6.0, 40.0)	100.0 (97.5, 100)	9.5 (0, 55.0)
Lymphovascular space invasion^b^	40 (44)	–	4 (25)	3 (20)	8 (56)	10 (67)	6 (40)	8 (100)	0 (0)
Lymph node involvement^b^	22 (24)	–	1 (6)	0 (0)	6 (38)	9 (60)	6 (40)	0 (0)	0 (0)

^a^Benign histologies included uterine fibroids (N = 12), ovarian serous cystadenoma (N = 5) endometriosis (N = 2), adenomyosis (N = 1), hydrosalpinx (N = 1), and ovarian fibroma (N = 1)

^b^Analysis limited to the 91 patients with cancer

The median total serum cfDNA concentration (prior to magnetic bead purification) was 69.2 ng/mL (IQR 37.4, 132.3). There was no significant association between total serum cfDNA concentration and body mass index (p = 0.17). There were no significant differences in total serum cfDNA concentration between control subjects (60.8 ng/mL IQR 5.3, 108.0) and women with cancer (70.7 ng/mL IQR 37.7, 138.2) (p = 0.33). There were also no significant differences in total serum cfDNA concentration across histologic subtypes (p = 0.06), grade of cancer (p = 0.46), stage of cancer (p = 0.33), percent myometrial invasion (p = 0.29), tumor size (p = 0.11), presence of LVSI (p = 0.12), or nodal involvement (p = 0.81).

**Fig. 1:**
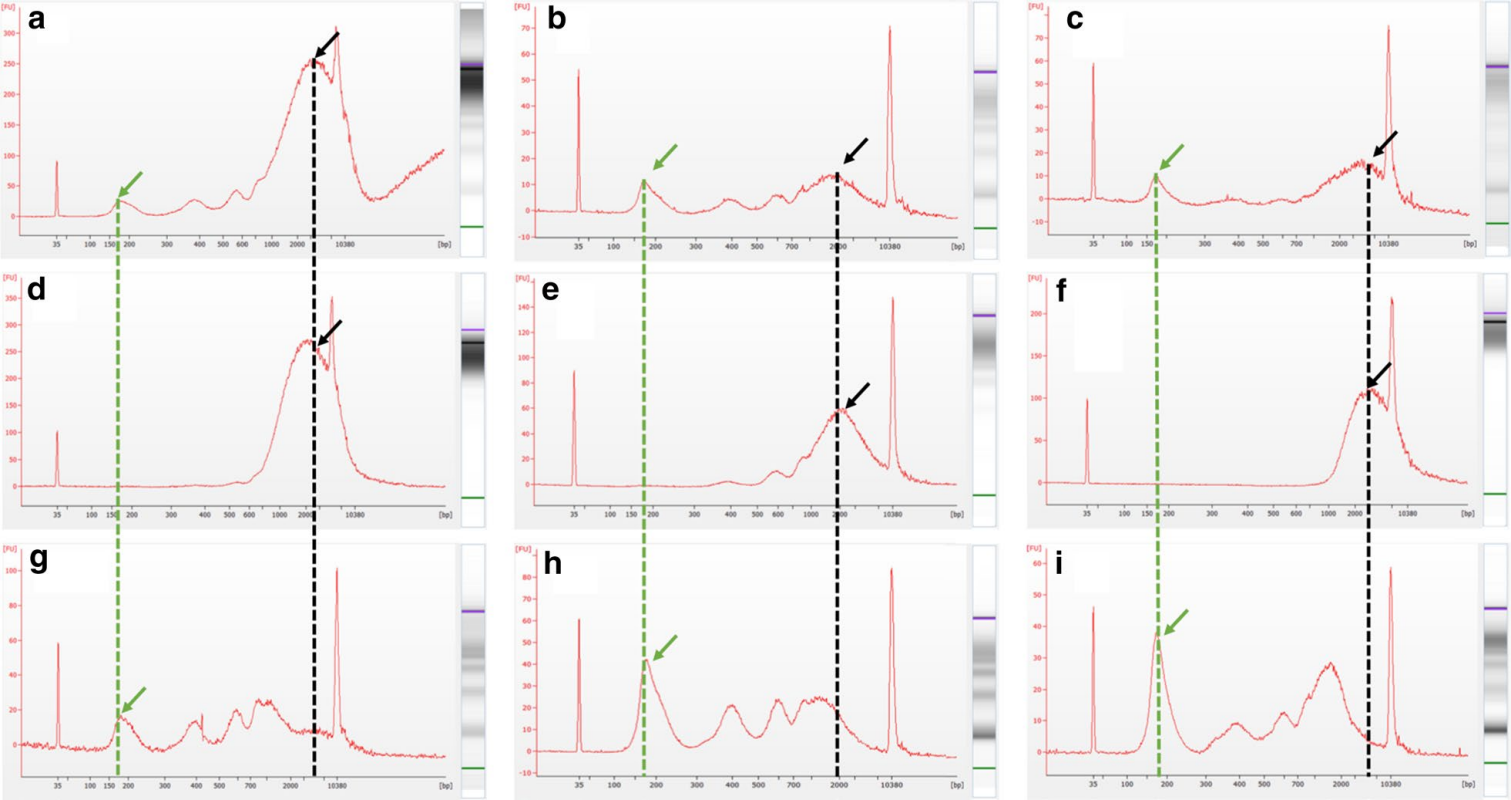
Representative bioanalyzer images of freshly isolated cfDNA from women with cancer (**a**–**c**) with contaminating high molecular weight DNA (marked with a black arrow and dotted line), the fraction of DNA from the same patients removed after the 0.5× SPRI AMPure bead purification step (**d–f**), and the resulting purified LMW cfDNA recovered after the 1.6× dilution SPRI AMPure bead purification (**g**–**i**). The desired cfDNA peak is marked with a green arrow and dotted line

The bioanalyzer profiles of fragment size distribution revealed a predominant peak of ~ 150–200 bp with a small additional peak around 350 bp and contamination of higher molecular weight bands around 10,380 bp (Fig. 1a–c). After purification with SPRI AMPure beads as described in the “Methods” section, the high-molecular weight DNA was removed (Fig. 1d–f) and the corresponding bands were no longer visible, leaving only the desired LMW cfDNA with peaks at 150 base pairs and additional peaks corresponding to nucleosomal (Fig. 1g–i).

**Fig. 2:**
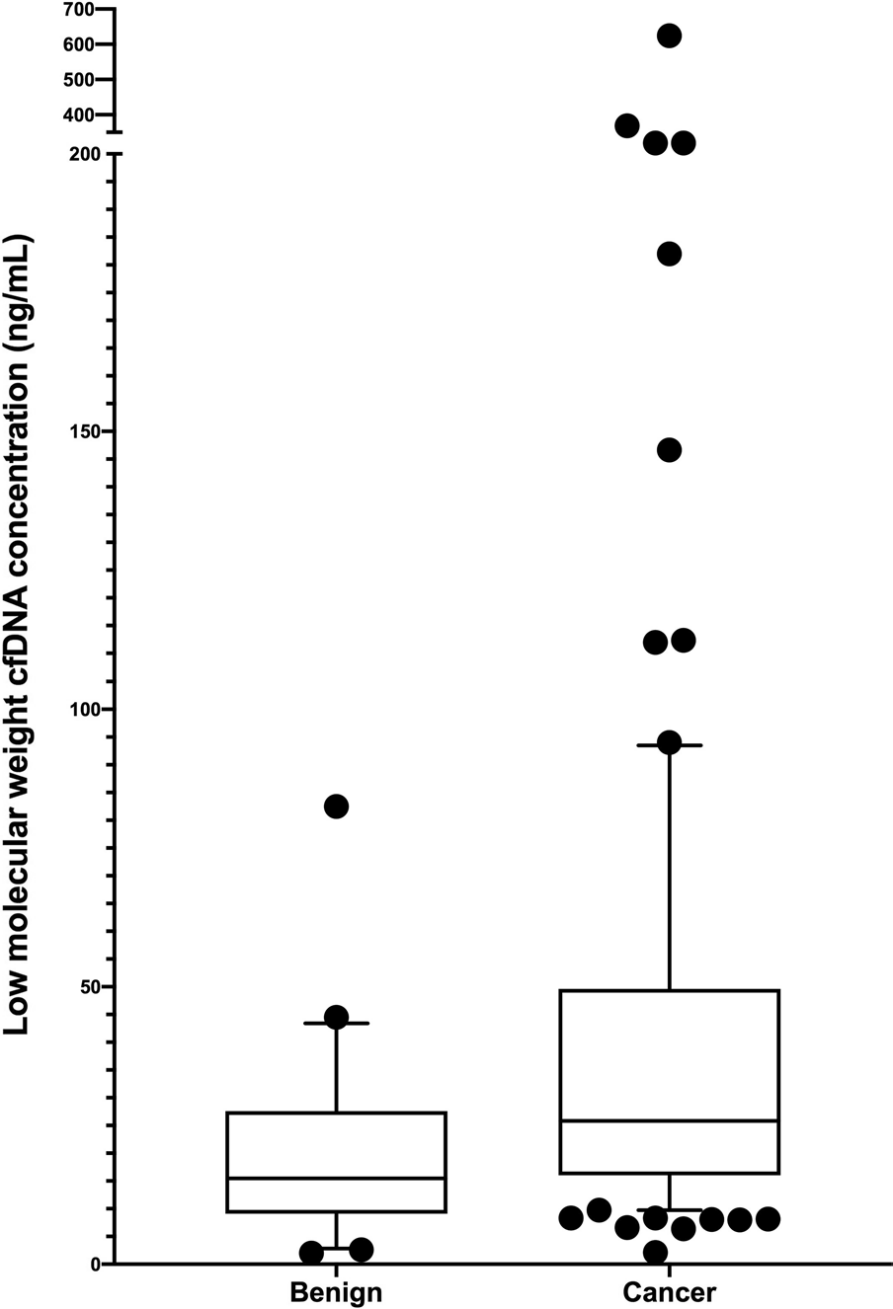
LMW cfDNA concentration stratified by cancer or benign disease

**Fig. 3:**
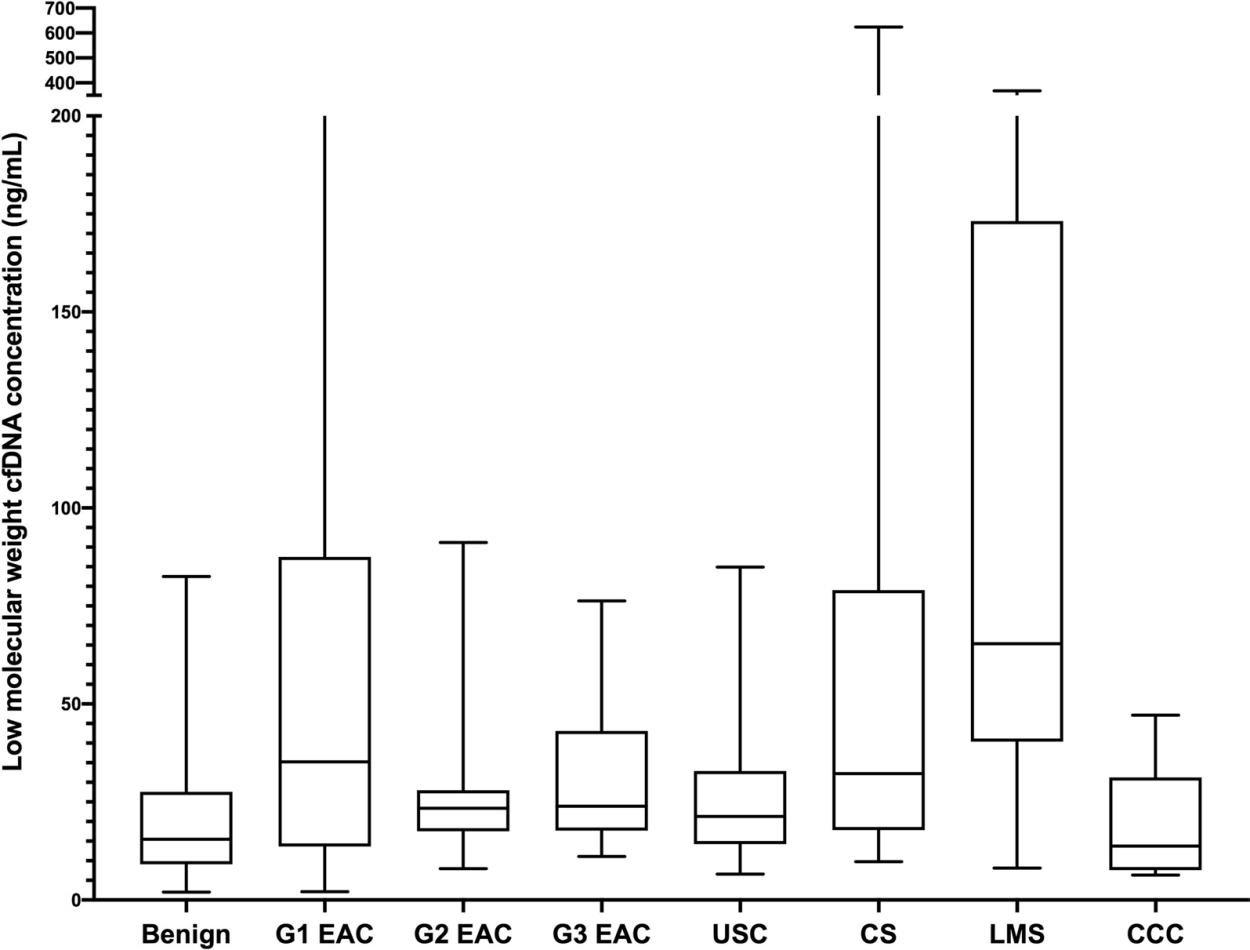
LMW cfDNA concentration stratified by histologic subtype

**Fig. 4:**
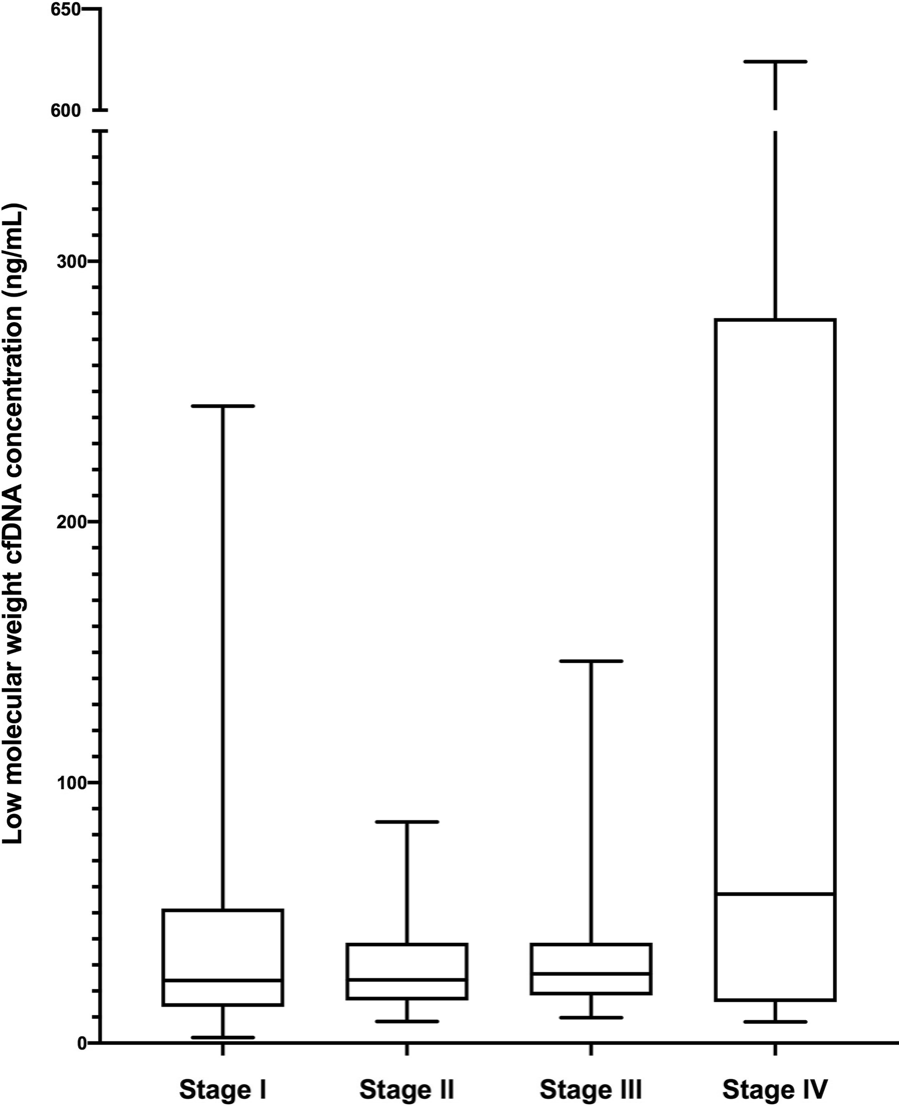
LMW cfDNA concentration stratified by stage of disease

The median serum LMW concentration (after bead purification) was 23.8 ng/mL (IQR 14.9, 44.4) for the entire cohort (Table 2). There was no significant association between LMW cfDNA and body mass index (p = 0.53). LMW cfDNA concentration was significantly higher in women with cancer (25.8 ng/mL IQR 16.0, 49.6) relative to controls (15.5 ng/mL, IQR 9.3, 25.8) (p < 0.01) (Fig. 2). Moreover, it was significantly higher in women with early stage (stages I and II) cancer compared to the controls (p < 0.01). Among cancer patients, there were also significant differences in LMW cfDNA concentration among histologic subtypes (p = 0.02). LMS (65.4 ng/mL IQR 45.7, 164.3) and CS (32.2 ng/mL IQR 17.9, 79.0) had the highest concentrations and EAC (25.2 ng/mL IQR 17.6, 44.4) had the lowest (Fig. 3). Within cancer patients, LMW cfDNA concentration was also significantly associated with cancer stage. Median LMW cfDNA concentration for patients with stage IV disease was significantly higher (57.2 ng/mL IQR 16.4, 246.1) than those with stage I–III disease (24.2 ng/mL IQR 16.0, 39.3) (p < 0.01) (Fig. 4). We also found marginally significant associations between LMW cfDNA concentration and the presence of LVSI (p = 0.06), and tumor size (p = 0.05), and no statistical significant association with the percent of myometrial invasion (p = 0.37), presence of nodal metastases (p = 0.39) or tumour grade (p = 0.49).

**Table 2:** Association of clinicopathologic variables with low-molecular weight (LMW) cfDNA concentration after bead purification and size-selection

	LMW cfDNA (ng/mL)	p-value
Full cohort (N = 113)	23.8 (14.9, 44.4)	
Benign (N = 22)	15.5 (9.3, 25.8)	0.006
Cancer (N = 91)	25.8 (16.0, 49.6)	
Histologic subtype		0.006
G1 EAC (N = 16)	35.3 (14.1, 83.5)	
G2 EAC (N = 15)	23.4 (17.6, 28.0)	
G3 EAC (N = 16)	23.9 (17.8, 41.9)	
UPSC (N = 15)	21.3 (14.3, 32.9)	
CS (N = 15)	32.2 (17.9, 79.0)	
LMS (N = 8)	65.4 (45.7, 164.3)	
CCC (N = 6)	13.8 (8.0, 26.0)	
Grade		0.04
Grade 1 (N = 16)	35.3 (14.1, 83.5)	
Grade 2 (N = 15)	23.4 (17.6, 28.0)	
Grade 3 (N = 60)	24.9 (16.1, 51.3)	
Stage of cancer		0.03
Stage I (N = 42)	24.0 (14.7, 47.1)	
Stage II (N = 16)	24.3 (16.8, 37.8)	
Stage III (N = 22)	26.6 (18.8, 36.6)	
Stage IV (N = 11)	57.2 (16.4, 246.1)	
Lymphovascular space invasion		0.04
Absent	21.6 (12.8, 39.3)	
Present	30.4 (17.8, 54.6)	
% Myometrial invasion		0.32
Tumor size		0.006
Lymph node status		
Negative	23.4 (14.4, 44.5)	0.39
Positive	30.4 (17.0, 44.4)	
Age		0.38
Body mass index		0.53
Race		
White	23.8 (14.9, 36.2)	0.66
Non-White	24.3 (14.8, 46.6)

**Fig. 5:**
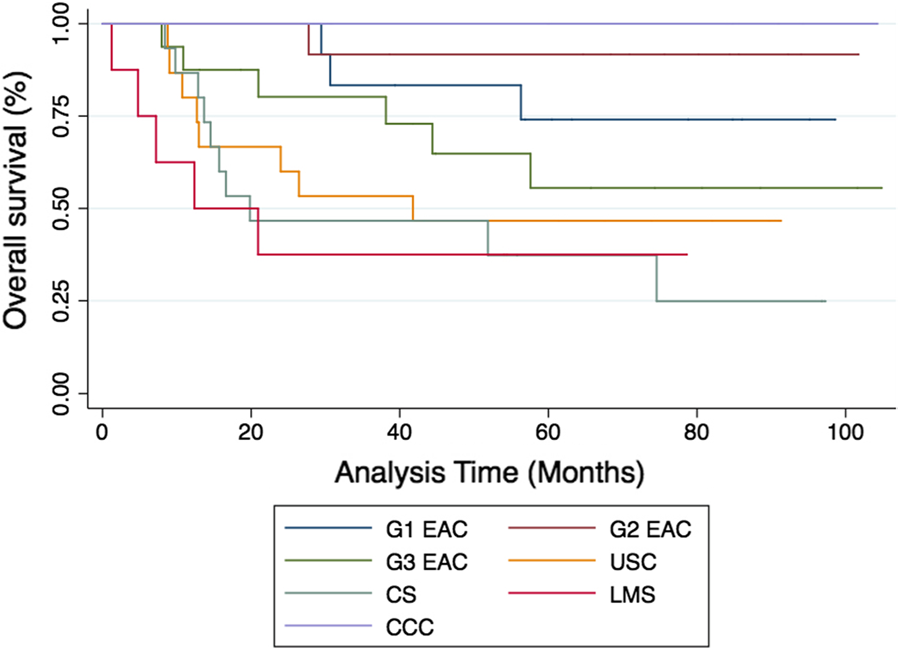
Kaplan-Meier survival estimates examining overall survival stratified by histologic subtype

**Fig. 6:**
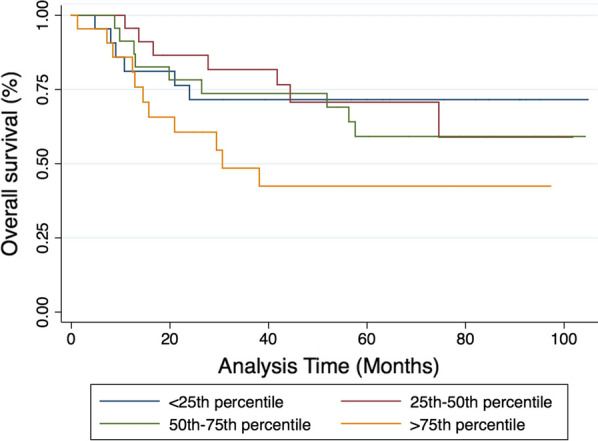
Kaplan-Meier survival estimates examining overall survival stratified by quartile of LMW cfDNA concentration

Over a median follow-up of 51.9 months, a total of 91 subjects contributed a total of 4574 person-months at risk. Thirty-three people within the cohort (36.3%) died of cancer, and the 24-month survival rate was 75%. Cox-proportional hazard modelling showed that the LMW cfDNA concentration is strongly associated with overall survival (p < 0.01), with hazard ratio at 1.53 as the concentration doubles. When analysing survival outcomes by quartile of LMW cfDNA concentration, patients with concentrations above the 75th percentile (> 49.6 ng/mL) had significantly worse survival than the remainder of subjects within the cohort (14.5 vs 41.8 months respectively, logrank p value = 0.04) (Fig. 5). Subjects with high-risk uterine histologies had worse overall survival (12.9 months) than those with endometrioid histologies (57.6 months) (p < 0.01) and subjects with leiomyosarcoma had the worst outcomes (4.8 months) (Fig. 5). When analysing survival outcomes by quartile of cfDNA concentration, those with concentrations above the 75th percentile (> 49.6 ng/mL) had significantly worse survival than the remainder of subjects within the cohort (14.5 vs 41.8 months respectively, logrank p-value = 0.04) (Fig. 6). This high-risk group included 22 women with various histologies including those with low grade EAC (N = 5), grade 2 EAC (N = 2), grade 3 EAC (N = 3), CS (N = 5), UPSC (N = 1) and LMS (N = 6). It also included women with both early stage (FIGO I–II, 54.5%) and late stage (FIGO III–IV, 45.5%) disease.

## Discussion

Our study demonstrates that LMW cfDNA concentration is significantly higher in women with uterine cancer relative to benign control subjects without cancer. It also shows that LMW cfDNA concentration correlates with indices of poor prognosis including aggressive histology, stage of disease, tumor size and presence of LVSI. Women with LMW cfDNA concentrations higher than 49.6 ng/mL had worse overall survival regardless of histologic subtype or stage of disease.

The finding that patients with cancer and those with metastatic disease have higher levels of cfDNA relative to healthy control subjects and patients without metastatic cancer respectively was first reported in 1977 [13]. Since that time, others have explored how the concentration of cfDNA varies with clinicopathologic features of tumors as well as in normal human physiology [14, 15]. The role of cfDNA concentration in uterine cancer classification and prognosis has been controversial. Cicchillitti et al. performed a quantitative analysis of cfDNA isolated from serum samples of 59 women with EAC and found a significant increase in this cohort relative to a control group without cancer. They also identified higher levels in women with grade 2 and grade 3 cancer compared to grade 1 samples [16]. They did not identify any differences in total cfDNA concentration when stratifying by cancer stage. Another group found increased levels of cfDNA in plasma of women with type II endometrial cancers relative to type 1 cancers [17], while others have demonstrated quantitative associations between cfDNA, uterine cancer grade and LVSI status, but not stage of disease [18]. A study by Tanaka et al. found differences in cfDNA concentration of women with uterine cancer relative to controls, but couldn’t find an association with grade of cancer, FIGO stage or treatment status [19].

It is challenging to draw definitive conclusions from the extant literature regarding cfDNA concentration and cancer status given that most studies use dramatically different cfDNA isolation techniques making direct comparisons problematic. A major confounding factor is likely a consequence of most investigators examining total cfDNA concentration levels rather than the fraction related to tumor apoptosis. The presence of high molecular weight DNA might bias results in many studies toward the null hypothesis. While the use of specialized cfDNA collection tubes designed to prevent cell lysis and the consequent release of high molecular weight DNA is becoming more common, many biorepositories are not equipped with these tubes giving rise to a wide range of technical variation.

Interestingly, in our study, total cfDNA concentration was not associated with any clinical variables, indicating that the proportion of tumor-related DNA with uterine cancer is likely very small and that our bead-based size selection protocol enriches for this cfDNA fraction. While we found significant differences in LMW cfDNA concentration by grade of cancer, concentration did not increase linearly with increasing grade. This was also reported by Cicchillitti et al. in which the average concentration in grade 3 uterine cancer was less than that of grade 2 cancer. While our study did not include mutational analysis of cfDNA specimens, our findings corroborate the findings of Bolivar et al. who found increased plasma DNA mutations related to presence of LVSI and increased tumor size [20].

Our findings corroborate the findings of other studies showing differences in cfDNA concentration relative to stage and aggressiveness of disease. Perhaps most interesting is our finding that women with LMS have increased cfDNA levels compared to those with other histologies. This is relevant, especially because there are no available imaging advances or non-invasive diagnostic modalities to reliably distinguish LMS from uterine fibroids. We suspect this is likely due to the fact that LMS often presents with advanced disease. However, we also determined that within the group of women who had the worst overall survival (> 75th percentile of LMW cfDNA concentration for the cohort) there was a broad mix of different histologies and stages of disease. While our study is too small to perform multivariate survival analysis, these findings suggest that LMW cfDNA concentration is associated with tumor aggressiveness beyond the risk conferred by stage of disease or specific histology.

The main benefit of our study is the inclusion of samples from a large cohort of racially-diverse women with many different uterine histologies and stages of disease. Because these samples were clinically well-annotated, we were able to compare LMW cfDNA concentration with clinical outcomes. On the other hand, we selected these samples a priori to ensure representation of multiple histologies and stages of disease and thus may not be reflective of the general population seeking care for uterine cancer. We suspect that the differences we see in LMW cfDNA and clinical outcomes is primarily determined by stage of cancer and extent of disease. However, our sample size is too limited to perform multi-variable modelling to control for confounding and determine the true driver behind increased cfDNA concentration in women with uterine cancer. While the sample size of the total cohort is robust, the subgroups included are too small to examine survival differences related to LMW cfDNA concentration stratified by histologic subtype. Larger studies specific to individual histologic subtypes would be necessary to answer this question.

## Conclusions

In summary, quantitative analysis of LMW cfDNA may hold promise, in combination with other traditional assays, as a clinically important adjunctive test in risk stratification of women with uterine cancer. Integration of cfDNA analysis is not yet widespread in the management of uterine malignancies and requires further validation.

## Data Availability

The datasets generated and/or analyzed during the current study are not publicly available due to infeasibility but are available from the corresponding author on reasonable request.

## Abbreviations

CCC: Clear cell carcinoma
cfDNA: Cell-free tumor DNA
CS: Carcinosarcoma
EAC: Endometrioid adenocarcinoma
FIGO: International federation of gynecology and obstetrics
LMW: Low-molecular weight
LMS: Leiomyosarcoma
LVSI: Lymphovascular space invasion
SPRI: Solid phase reversible immobilization beads
UPSC: Uterine papillary serous carcinoma
